# Uterine transcriptome analysis reveals mRNA expression changes associated with the ultrastructure differences of eggshell in young and aged laying hens

**DOI:** 10.1186/s12864-020-07177-7

**Published:** 2020-11-09

**Authors:** Jia Feng, Hai-jun Zhang, Shu-geng Wu, Guang-hai Qi, Jing Wang

**Affiliations:** Laboratory of Quality & Safety Risk Assessment for Animal Products on Feed Hazards (Beijing) of the Ministry of Agriculture & Rural Affairs, Feed Research Institute, Chinese Academy of Agricultural Sciences, Beijing, 100081 China

**Keywords:** Laying hen, Late laying period, Eggshell quality, Ultrastructure, Uterus, Transcriptome

## Abstract

**Background:**

Lower eggshell quality in the late laying period leads to economic loss. It is a major threat to the quality and safety of egg products. Age-related variations in ultrastructure were thought to induce this deterioration. Eggshell formation is a highly complex process under precise regulation of genes and biological pathways in uterus of laying hens. Herein, we evaluated the physical, mechanical and ultrastructure properties of eggshell and conducted RNA sequencing to learn the transcriptomic differences in uterus between laying hens in the peak (young hens) and late phase (aged hens) of production.

**Results:**

The declined breaking strength and fracture toughness of eggshell were observed in aged hen group compared to those in young hen group, accompanied with ultrastructure variations including the increased thickness of mammillary layer and the decreased incidence of early fusion. During the initial stage of eggshell formation, a total of 183 differentially expressed genes (DEGs; 125 upregulated and 58 downregulated) were identified in uterus of laying hens in the late phase in relative to those at peak production. The DEGs annotated to Gene Ontology terms related to antigen processing and presentation were downregulated in aged hens compared to young hens. The contents of proinflammatory cytokine IL-1β in uterus were higher in aged hens relative to those in young hens. Besides, the genes of some matrix proteins potentially involved in eggshell mineralization, such as ovalbumin, versican and glypican 3, were also differentially expressed between two groups.

**Conclusions:**

Altered gene expression of matrix proteins along with the compromised immune function in uterus of laying hens in the late phase of production may conduce to age-related impairments of eggshell ultrastructure and mechanical properties. The current study enhances our understanding of the age-related deteriorations in eggshell ultrastructure and provides potential targets for improvement of eggshell quality in the late laying period.

**Supplementary Information:**

**Supplementary information** accompanies this paper at 10.1186/s12864-020-07177-7.

## Background

Great economic loss caused by lower eggshell quality is a major concern for egg industry and this problem can be more serious in the late laying period [1–3]. It has been estimated that the incidence of cracked and broken eggs during the late phase of production could reach as high as 12–20% [4], which is one of the key obstacles for extending the laying cycle of commercial flocks [5]. Eggshell is a highly ordered structure comprising membranes (inner and outer), mammillary layer, palisade layer, vertical crystal layer and cuticle. The key roles of ultrastructure have been increasingly recognized in determining shell mechanical properties and quality [6, 7]. Eggshell in the late phase of production possessed lower breaking strength, accompanied with a greater variability in structural properties such as thickness, grain morphology and crystal texture [7]. Besides, the percentages of abnormal structure such as type B mammillae and late fusion in eggshell from aged hens were higher than those from young hens [8], which was thought to contribute to the impairment of mechanical properties. Therefore, age-related abnormalities in ultrastructure could be one reasonable explanation for the compromised shell quality in the late laying period.

The ultrastructure results from the sequential precipitation of mineral carbonate and organic matrix during three stages of mineralization (the initiation, rapid growth and the termination) [9]. Organic matrix plays a crucial role in modulating ultrastructure and mechanical properties of eggshell, as confirmed by in vitro and in vivo observations and by analogies with other biominerals [10, 11]. Eggshell formation is a highly complex process under precise control of genes and biological pathways in uterus [12, 13]. More than 37 ion transporter genes (such as calbindin, ATP2A3, TRPV6 and CA2), which participate in the supply of ions and minerals for eggshell formation, have been identified to establish the uterine ion transport model [14]. A recent transcriptome analysis indicated some novel genes (such as ATP2C2, stanniocalcin 2 and calcitonin-related polypeptide B) and three canonical pathways including calcium transport I, cAMP-mediated signaling and cardiac β-adrenergic signaling potentially involved in regulating eggshell formation [15]. Furthermore, the genetic bases of ultrastructure traits and mechanical properties of eggshell have also been extensively explored. For example, genetic variations of ovocleidin-116, ovotransferrin and secreted phosphoprotein 1 were linked to the decreased eggshell quality [16]. Matrix protein genes such as ovalbumin, ovocleidin-116 and ovocalyxin-32 were associated with shell thickness and mammillary layer thickness [17], while ABCC9, ITPR2, KCNJ8 and WNK1, which are involved in ion transport, were suggested to be the key genes modulating eggshell thickness and effective thickness [18]. Hence, deteriorations in eggshell ultrastructure and quality in the late phase of production were speculated to be associated with age-related dysregulation of gene expression in uterus. It was supported by the findings that the expression of genes encoding ion transporters and matrix proteins (ATP2A2, SCNN1G, CA2 and ovocalyxin-36) changed with the age of laying hens [19]. Age-related changes in the expression of matrix proteins (ovalbumin, ovotransferrin and ovocleidin-17) in uterus of laying hens were suggested by their varying concentration in eggshell [20, 21]. Besides, a destructed uterine structure, the reduced gland density [22] and the fibrosis and atrophy of the endometrium [8] of aged hens probably caused by sustained laying behavior would contribute to the uterine hypofunction of protein synthesis, ion transport and immune defense. However, a comprehensive understanding of uterine functional differences in terms of eggshell formation between young and aged hens at the transcriptome level remains obscure.

This study was performed to evaluate age-related changes in eggshell quality and ultrastructure and thus determine the crucial ultrastructure layer as well as its corresponding formation stage responsible for the declined eggshell quality in the late phase of production. Then, RNA sequence was employed to investigate transcriptomic differences in uterus between laying hens in different laying periods at a specific stage of eggshell formation. Our study may contribute to the identification of important genes associated with age-related deterioration in eggshell quality and providing potential targets to improve eggshell quality in the late phase of production.

## Results

### The physical and mechanical properties of eggshell

The contents of major inorganic materials (calcium and phosphorus) and total matrix protein did not change significantly (*P* > 0.05) with hen age (Table 1). Egg and shell weight were higher (*P* < 0.05) in the aged hens relative to the young hens, whereas shell thickness and ratio were similar (*P* > 0.05) for these two groups. In terms of shell mechanical properties, breaking strength and fracture toughness of eggshell from aged hens were significantly lower (*P* < 0.05) than those of young ones.

**Table 1:** Comparison of chemical composition, physical and mechanical properties of eggshell between young (42 wk. of age) and aged hen groups (72 wk. of age)

Items	Young hen	Aged hen	*P*-value
Calcium content (mg/g)	370.62 ± 19.72	344.68 ± 33.96	0.083
Phosphorus content (μg/g)	1.11 ± 0.12	1.00 ± 0.17	0.169
Matrix protein content (mg/g)	13.72 ± 1.18	13.32 ± 1.18	0.507
Shell thickness (mm)	0.37 ± 0.02	0.39 ± 0.02	0.234
Egg weight (g)	64.42 ± 0.54	65.83 ± 1.12	0.006
Shell weight (g)	6.37 ± 0.05	6.49 ± 0.10	0.008
Shell ratio (%)	9.89 ± 0.05	9.86 ± 0.04	0.244
Breaking strength (N)	46.32 ± 1.64	36.85 ± 2.74	< 0.001
Stiffness (N/mm)	74.88 ± 1.91	75.39 ± 3.16	0.702
Elastic modules (N/mm^2^)	7636.34 ± 133.61	7583.75 ± 261.38	0.620
Fracture toughness (N/mm^3/2^)	573.11 ± 40.29	456.35 ± 38.75	< 0.001

Data are the mean of 8 replicates with 24 eggs each (another 12 eggs of each replicate for the measurement of stiffness, elastic modules and fracture toughness)

### Age-related deterioration in eggshell ultrastructure

**Fig. 1:**
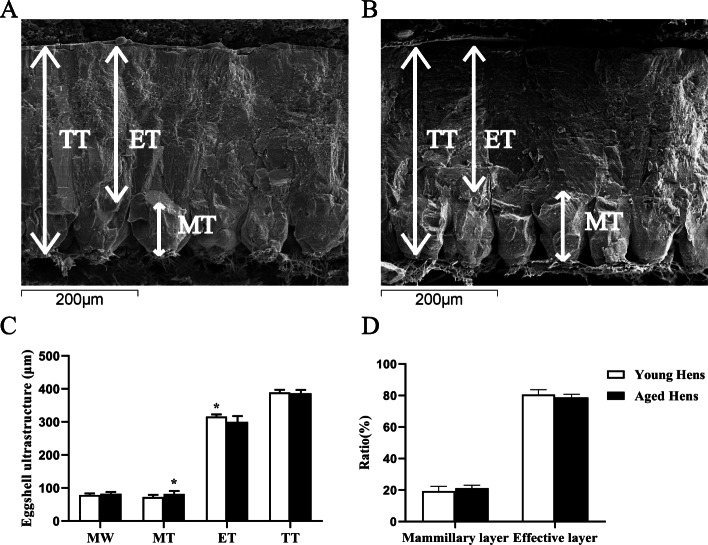
The eggshell ultrastructure in young (**a**; 42 wk. of age) and aged hen groups (**b**; 72 wk. of age; scanning electron microscope images, magnification, 200×). The ultrastructural characteristics (**c**&**d**) of eggshell in young and aged hen groups. MW, mammillary width; MT, mammillary layer thickness; ET, effective layer thickness; TT, total thickness. Data are the mean of 8 replicates with 24 eggs each. Asterisk represents a significant difference (*P* < 0.05) between groups

**Fig. 2:**
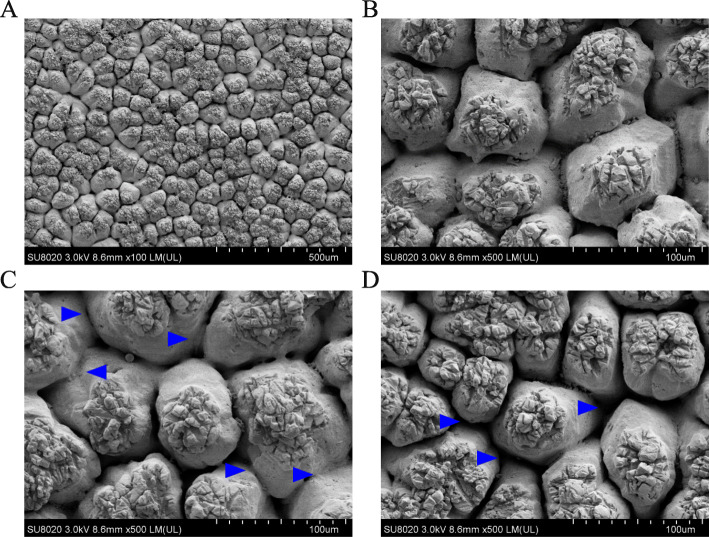
Scaning electron micrograghs showing a mammillary view (**a**; magnification, 100×) of eggshell, normal mammillary body (**b**; 500×), early fusion of mammillary knobs (**c**; 500×) in young hen group (42 wk. of age) and late fusion of mammillary knobs (**d**; 500×) in aged hen group (72 wk. of age)

There was a significant increase in the thickness of mammillary layer (72.87 ± 6.47 vs. 81.98 ± 9.12, young vs. aged hens, *P* = 0.037), but a significant decline (*P* < 0.05) in effective thickness (316.38 ± 6.20 vs. 300.26 ± 17.64, *P* = 0.029) of aged hens relative to those of young ones (Fig. 1). No differences (*P* > 0.05) were observed between groups in mammillary knob width, total shell thickness, the ratio of mammillary and effective layer. Most ultrastructure variants in mammillary layer were not affected by hen age (*P* > 0.05), while there was a reduction in the incidence of early fusion (*P* < 0.05) in mammillary layer of eggshell in aged hens as compared to young ones (Table 2 & Fig. 2). As a result, total score for mammillary variants was higher (*P* < 0.05) in aged hen group relative to young one.

**Table 2:** Comparison of ultrastructural variations in mammillary layer of eggshell between young (42 wk. of age) and aged hen groups (72 wk. of age)

Ultrastructural variation	Young hen	Aged hen	*P*-value
Mammillae density	224.53 ± 13.48	221.92 ± 11.80	0.686
Confluence	2.59 ± 1.04	2.97 ± 0.91	0.456
Type B	1.72 ± 0.76	1.94 ± 0.40	0.483
Type A	1.13 ± 0.13	1.16 ± 0.19	0.705
Aragonite	1.03 ± 0.09	1.16 ± 0.19	0.117
Early fusion	1.72 ± 0.62	2.59 ± 0.92	0.042
Late fusion	3.13 ± 1.13	4.22 ± 1.16	0.077
Cuffing	2.97 ± 1.39	3.28 ± 0.75	0.583
Pitted	1.13 ± 0.35	1.25 ± 0.46	0.554
Caps	2.66 ± 0.87	3.06 ± 0.79	0.343
Total score	19.06 ± 2.98	22.63 ± 2.68	0.025

Data are the mean of 8 replicates with 24 eggs each

### Proinflammatory cytokine contents in uterus tissue

The content of proinflammatory cytokine IL-1β in uterus in the aged hens was higher (*P* < 0.05, Table 3) than that in the young hens, while IL-6 and IFN-γ contents were not affected (*P* > 0.05) by hen age.

**Table 3:** Comparison of the contents of proinflammatory cytokines in uterine mucosa between young (42 wk. of age) and aged hen groups (72 wk. of age)

Items	Young hen	Aged hen	*P*-value
IL-1β (pg/mg protein)	7.47 ± 0.57	8.17 ± 0.47	0.019
IL-6 (pg/mg protein)	0.33 ± 0.04	0.36 ± 0.05	0.155
IFN-γ (pg/mg protein)	0.78 ± 0.07	0.82 ± 0.09	0.348

Data are the mean of 8 replicates with 2 birds each (one sample from the aged hen group was rejected due to no egg with incomplete shell present in hen oviduct)

### Identification of differentially expressed genes (DEGs) in uterus between two groups

**Fig. 3:**
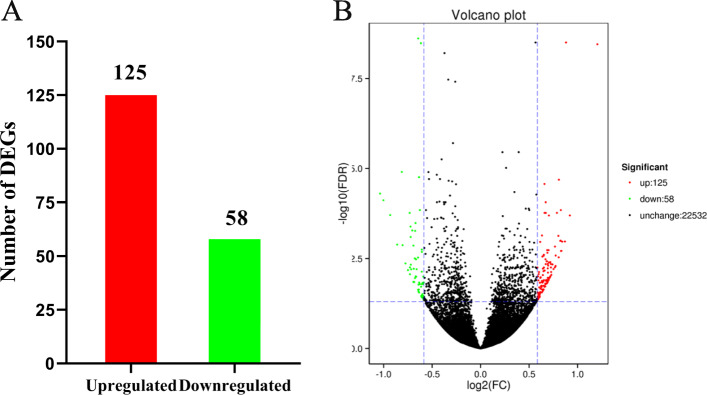
The differentially expressed genes (DEGs; |fold change| > 1.5 at a false discovery rate (FDR) < 0.05; **a**) and the differentially expressed volcano diagram (**b**) of uterus in aged hen group (72 wk. of age) relative to young hen group (42 wk. of age)

**Fig. 4:**
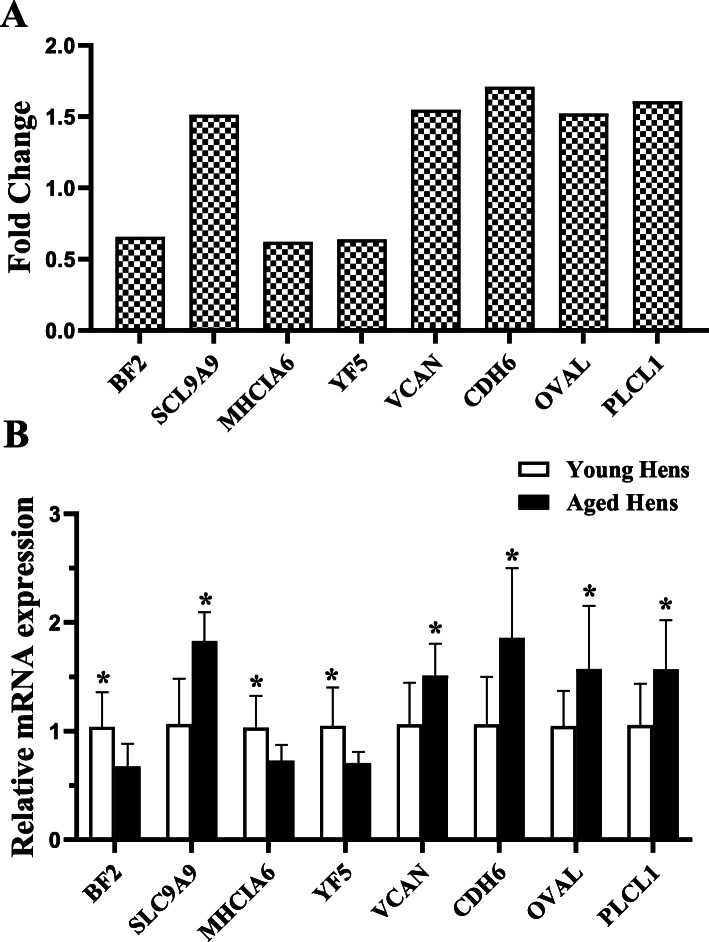
Validation of the differentially expressed genes (DEGs) by RT-qPCR. **a** Fold change of transcriptome results for DEGs in aged hen group (72 wk. of age) relative to young hen group (42 wk. of age). **b** Relative mRNA expression of the DEGs. BF2, MHC class I antigen BF2; SLC9A9, solute carrier family 9 member A9; MHCIA6, major histocompatibility complex, class I, A6; YF5, MHC class I α-chain; VCAN, versican; CDH6, cadherin-6; OVAL, ovalbumin; PLCL1, phospholipase C like 1. Values are means and standard deviations represented by vertical bars and the values with asterisks mean significant difference between groups (*P* < 0.05)

A total of 739,940,606 (220.78 Gb of clean data) clean reads were generated from the thirty-one libraries divided into two groups, with more than 20 million clean reads from each sample (Additional file 1). About 37–52 × 10^6^ reads (88–92% of the total raw reads) were uniquely mapped to *Gallus gallus* genome. More than 91% bases had a quality score of ≥ Q30 and the GC contents of the libraries were ranged from 47.95 to 51.15%, close to 50%, indicating a reliable quality of RNA sequence results. A total of 183 DEGs were identified in uterus between the young and aged hen groups (Fig. 3a & Additional file 2). Among these DEGs, there were 125 significantly up-regulated and 58 significantly down-regulated genes in the aged hens relative to the young ones and the differences in the gene expression profile between groups were visualized in volcano plot (Fig. 3b). We selected 8 genes for quantitative real-time (qRT) -PCR to verify the transcriptome data, and the relative RNA expression was consistent with the results from transcriptome analysis (Fig. 4).

### Functional annotation and pathway enrichment analysis of DEGs between two groups

**Fig. 5:**
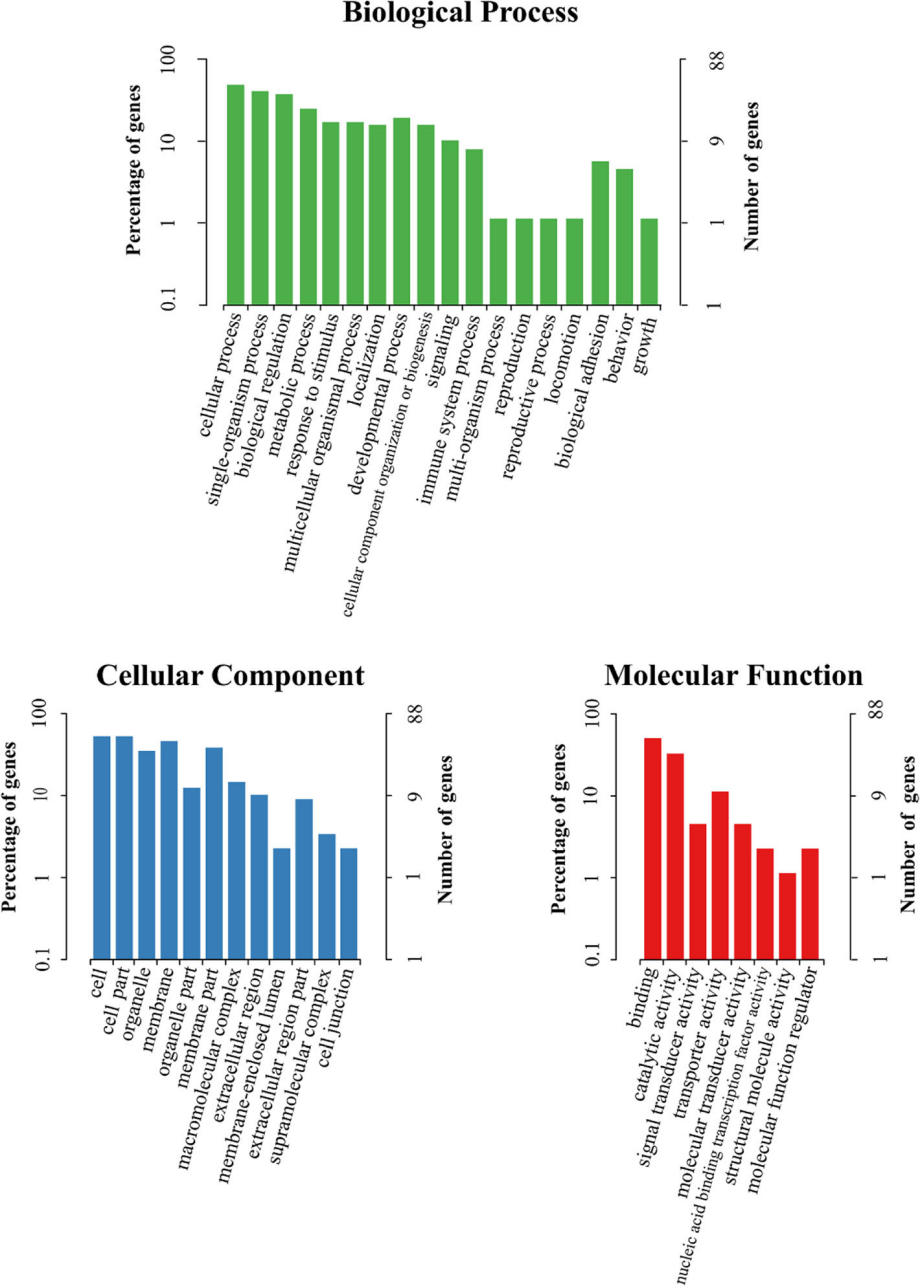
Gene Ontology classification analysis of differentially expressed genes of uterus in age hen group (72 wk. of age) relative to young hen group (42 wk. of age)

**Fig. 6:**
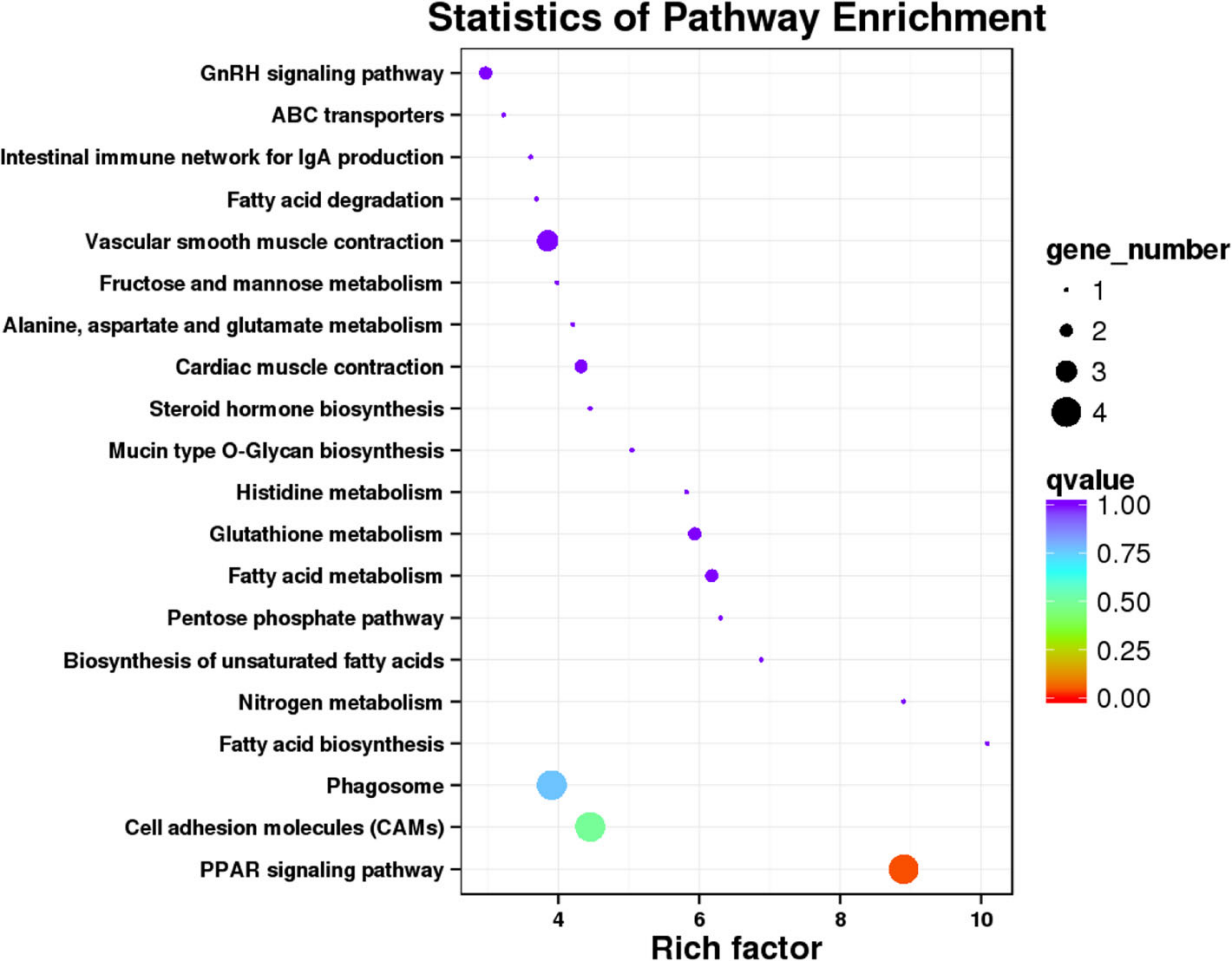
Pathway analysis of differential expressed genes of uterus in aged hen group (72 wk. of age) relative to young hen group (42 wk. of age). The differential expressed genes in aged hen group relative to young hen group were only enriched (*Q* < 0.05) in the peroxisome proliferator-activated receptor (PPAR) signaling pathway (rich factor = 8.90)

Gene Ontology (GO) and Kyoto Encyclopedia of Genes and Genomes (KEGG) enrichment analysis were performed to obtain valuable information for functional prediction of DEGs. In total, the DEGs between groups were functionally distributed into 13 COG (Clusters of Orthologous Groups) categories (Additional file 3). Among them, the greatest number of DEGs were assigned to the category of general function prediction only (31.91%), followed by the category of amino acid transport and metabolism (12.77%), secondary metabolites biosynthesis, transport and catabolism (10.64%), posttranslational modification, protein turnover, chaperones (8.51%), cytoskeleton (8.51%), and inorganic ion transport and metabolism (6.38%). The DEGs were annotated into three major functional categories (Fig. 5) including biological process, cellular component and molecular function. The most enriched terms in the category of biological process were cellular process, single-organism process and biological regulation. Cell, cell part and organelle were most enriched in the category of cellular component and binding, catalytic activity and signal transducer activity were most enriched in the category of molecular function. The DEGs in the aged hen group relative to the young hen group were only enriched in the pathway of peroxisome proliferator-activated receptor (PPAR) signaling pathway (rich factor (RF) = 8.90, Fig. 6). In the PPAR signaling pathway, stearoyl-CoA desaturase (SCD, fold change (FC) =0.61), acyl-CoA synthetase bubblegum family member 1 (ACSBG1, FC = 1.51), apolipoprotein C3 (APOC3, FC = 0.64), and fatty acid binding protein 5 (FABP5, FC = 0.66) were differentially expressed between two groups.

### GO clustering analysis of DEGs between two groups

The DEGs were primarily enriched in the following terms within the category of biological process (Table 4): antigen processing and presentation of peptide antigen via major histocompatibility complex (MHC) class I, positive regulation of T cell mediated cytotoxicity, regulation of catabolic process and antigen processing and presentation. Within the category of cellular component, the clusters were enriched in MHC class I, membrane, integral component of membrane, mitochondrion and cytoplasmic part. In terms of the category of molecular function, the enriched terms were peptide antigen binding, catalytic activity, identical protein binding, transferase activity and antigen binding. Since DEGs were predominantly enriched in the GO terms associated with immune response, further analysis was conducted on the DEGs in relation to immune response and immune defense, as well as the DEGs involved in eggshell formation (Table 5). Ten DEGs encoding matrix proteins or calcium transport proteins were suspected to play a role in eggshell formation. Among the immune-related DEGs, MHC class I antigen BF2 (BF2), MHC class I A molecules 5, 6 (MHCIA5 and MHCIA6) and MHC class I α-chain (YF5) in association with antigen processing and presentation were all downregulated in aged hens relative to young ones. The expression of ABCA13 and LOC107056420 related to defense mechanism were all upregulated in the aged hen group as compared to young hen group.

**Table 4:** Gene oncology (GO) clustering analysis of differentially expressed genes (DEGs; |fold change| > 1.5 at a false discovery rate < 0.05)

GO Terms	GO.ID	Number of DEGs	*P*-value
**Biological Process**			
antigen processing and presentation of peptide antigen via MHC class I	GO:0002474	4	< 0.001
positive regulation of T cell mediated cytotoxicity	GO:0001916	4	< 0.001
regulation of catabolic process	GO:0009894	2	0.005
antigen processing and presentation	GO:0019882	5	0.006
protein ubiquitination involved in ubiquitin-dependent protein catabolic process	GO:0071947	2	0.011
protein modification by small protein conjugation or removal	GO:0070647	5	0.016
cellular protein modification process	GO:0006464	10	0.043
protein modification process	GO:0036211	10	0.043
macromolecule modification	GO:0043412	10	0.045
**Cellular Component**			
MHC class I protein complex	GO:0042612	4	< 0.001
membrane	GO:0016020	41	< 0.001
integral component of membrane	GO:0016021	25	< 0.001
mitochondrion	GO:0005739	4	0.001
cytoplasmic part	GO:0044444	16	0.004
endomembrane system	GO:0012505	8	0.016
membrane part	GO:0044425	34	0.026
endosome	GO:0005768	3	0.029
intrinsic component of membrane	GO:0031224	27	0.042
extracellular matrix	GO:0031012	3	0.043
**Molecular Function**			
peptide antigen binding	GO:0042605	4	< 0.001
catalytic activity	GO:0003824	29	< 0.001
identical protein binding	GO:0042802	2	0.004
transferase activity	GO:0016740	11	0.005
antigen binding	GO:0003823	4	0.005
calcium ion binding	GO:0005509	5	0.008
adenyl ribonucleotide binding	GO:0032559	3	0.015
ATP binding	GO:0005524	3	0.015
ion binding	GO:0043167	31	0.018
adenyl nucleotide binding	GO:0030554	3	0.018
oxidoreductase activity	GO:0016491	3	0.020
oxidoreductase activity, acting on paired donors, with incorporation or reduction of molecular oxygen	GO:0016705	2	0.026
voltage-gated calcium channel activity	GO:0005245	2	0.047

**Table 5:** Analysis of differentially expressed genes (|fold change| > 1.5 at a false discovery rate < 0.05) in association with matrix protein, calcium transport, immune response and defense mechanism

Potential function	Gene (Fold change)	Annotation
Matrix protein	VCAN (1.55)	Versican, chondroitin sulfate proteoglycan, calcium ion binding
	CDH6 (1.71)	Cadherin 6, calcium binding, in association with the physiological control of bone remodeling
	LOC107056462 (1.61)	Alpha-actinin-1-like, calcium binding
	PLCL1 (1.61)	Phospholipase C like 1, calcium binding
	OVAL (1.52)	Ovalbumin, regulate shell structure and present in eggshell
	GPC3 (0.64)	Glypican 3, regulate the activity of bone morphogenetic proteins and promote bone formation
	DMP1 (0.66)	Dental matrix protein-1, in association with tissue mineralization
Calcium transport	CACNA1C (1.79)	Voltage-dependent L-type calcium channel subunit alpha-1C, ion transport protein, voltage-gated calcium channel activity
	CACNA1D (1.68)	Voltage-dependent L-type calcium channel subunit alpha-1D, ion transport protein, voltage-gated calcium channel activity
	NMUR2 (1.52)	Thyrotropin-releasing hormone receptor, calcium ion transport
Immune response	BF2 (0.66)	MHC class I antigen BF2, antigen processing and presentation
	MHCIA5 (0.58)	Major histocompatibility complex, class I, A5, antigen processing and presentation
	MHCIA6 (0.62)	Major histocompatibility complex, class I, A6, antigen processing and presentation
	YF5 (0.64)	MHC class I antigen YF5, antigen processing and presentation
	LOC101748499 (1.64)	Class II histocompatibility antigen, B-L beta chain-like, immune response
	IL7 (1.63)	Interleukin 7, a cytokine important for B and T cell development
	C7 (1.61)	Complement C7, as part of the terminal complement pathway of the innate immune system
	KCTD7 (1.71)	Potassium channel tetramerization domain containing 7, negative regulation of inflammation
Defense mechanism	ABCA13 (1.65)	ATP-binding cassette, sub-family A, membrane 13, defense mechanism
	LOC107056420 (1.50)	Intestinal mucin-like protein, defense mechanism

## Discussion

The breaking strength of eggshell declines during the production period of laying hens [23, 24], which was also observed in this study. A recent study evaluated shell quality of eggs sampled every 5 wks, from 30 to 81 wk. of hen age and the results showed that eggshell from the peak phase (30–53 wk. of age) and the late phase (58–81 wk. of age) of production could be distinguished in terms of egg physical and eggshell quality attributes [23]. The decreased shell quality in the late phase of production could be attributed to the increased egg size but no proportional increases in shell weight. This was consistent with our results that a reduction in the contents of calcium in eggshell concurrent with an increase in the weight of egg and eggshell were observed in aged hen group. Besides, it has been suggested that age-associated deteriorations in ultrastructure would contribute to the compromised mechanical properties of eggshell in the late laying period. The variability of ultrastructure quality attributes such as thickness, grain morphology and crystal texture [7], and the occurrence of abnormal structures including type A and B mammillae and mammillary alignment in eggshell [8] were reported to increase with the advance of hen age. In the current study, eggshell ultrastructure in aged hens was characterized by thicker mammillary layer, thinner effective layer and less frequency of early fusion. The thickness and proportion of effective layer were the major ultrastructural characteristics determining shell strength and a reduction in its thickness could compromise shell strength conducing to a higher incidence of breakage [25]. Stress concentrations would occur more frequently and rapidly in the site where the fusion of adjacent mammillary columns was delayed or where structural abnormalities arose. Less early fusion of mammillae in aged hen group suggested a weak binding connection between mammillary knobs, where a crack would propagate easily through shell and outwards from the force points, and therefore compromised its mechanical properties [25, 26]. These changes might further induce negative effects on the structure and quality of following layers [27]. Therefore, ultrastructural variations in eggshell especially in the mammillary layer, may trigger the impairment of shell mechanical properties in aged hen group. RNA sequencing analysis was employed to reveal differences between young and aged hen groups in transcriptome profile of uterus during the initial phase (8.5 h post-oviposition (PO)), corresponding to the stage of mammillary layer formation.

Structural formation is controlled by precise interactions between mineral and organic precursors, which are secreted by specialized oviduct cell populations under gene regulation [13, 28]. Calcium is the major component of eggshell and many calcium transport genes (such as calbindin, ATP2B1, 2 and TRPV6) have been identified to be involved in the supply of calcium for eggshell formation. In this study, there were three calcium transport-related DEGs including thyrotropin-releasing hormone receptor, calcium voltage-gated channel subunit alpha1 C and 1 D (CACNA1C and CACNA1D) in uterus between young and aged hen groups. CACNA1C and CACNA1D mediate the entry of calcium ions into excitable cells and are also involved in a variety of calcium-dependent processes and MAPK signaling pathways [29]. Besides, solute carriers were proposed to actively involve in ions and minerals transport for eggshell formation in the uterus [15, 30]. The overexpression of SLC9A9 encoding protein would cause excess leak of protons from the recycling endosomes leading to more alkaline endosomes [31]. In our study, the relative higher expression levels of SLC9A9 in the aged hens as compared to the young hens may indicate an age-related change of proton exclusion and Na^+^ influx in the uterus. However, their functions in eggshell formation have never been reported and need further investigation.

Calcium-binding property is the prerequisite for matrix proteins to participate in biomineralization. Numerus matrix proteins have been identified to play a regulatory role in the initial stage of eggshell formation [10, 14, 32]. Ovalbumin, one of the most dominant matrix proteins in this period, could serve as an effective stabilization agent for transient precursors and prevent undirected mineralization [33]. It would preferentially interact with the obtuse rather than the acute steps on the (104) calcite face and thus crystals from the nucleation site could only grow in specific directions. Besides, this effect is closely dependent on its concentration and it would be striking at a higher concentration [34]. In this study, the upregulated expression of ovalbumin with age, consistent with previous observation in shell extracts [21], suggested that calcite crystals would grow in specific and limited directions rather than in all directions, presumptively leading to the delay of mammillary fusion and a thicker mammillary layer. This was supported by the findings that the variations in ovalbumin gene were associated with mammillary layer thickness and breaking strength of eggshell through candidate gene association analysis [17]. Phospholipase C like 1 (PLCL1) protein can inhibit inositol 1,4,5-trisphosphate (IP3)-mediated calcium signaling regulating mechanical sensing of bone cells, supporting its important role in bone formation [35]. Versican (VCAN) encoding a large chondroitin sulfate proteoglycan, could interact with other matrix/cell surface molecules to facilitate establishment or maintenance of early joint interzone structure [36]. Cadherin-6 (CDH6), a single chain transmembrane glycoprotein, can interact selectively and non-covalently with calcium ions and the expression of CDH6 in mouse stromal cells was reported to be associated with the physiological control of bone remodeling [37]. Therefore, the increased expression of ovalbumin, PLCL1, VCAN and CDH6 with the resultant upregulations of GO terms of calcium ion binding might suggest a disordered regulation of matrix proteins, resulting in ultrastructural defects and poor quality of eggshell in the aged hen group.

Proteoglycans (keratan and dermatan sulfate proteoglycans) have an affinity for calcium and were thought to affect the nucleation and assemble of calcite crystals [38]. Glypicans (GPC), belonging to the heparan sulphate proteoglycans, were suspected to be involved in biomineralization. The expression of GPC4 in the shell gland of hens was only found during shell calcification and regulated by egg mechanical strain [39]. GPC3 was regarded as a critical molecule to regulate the activity of bone morphogenetic proteins and promote bone formation. The deletion of GPC3 gene was reported to cause a disorder of bone mineralization and malformation in ribs and skull sutures [40]. In addition, bony suture fusion was associated with the altered expression of GPC3. Accordingly, the downregulations of GPC3 in aged hen group might induce abnormal fusion of mammillary knobs, presumptively contributing to the compromised structure and quality of eggshell. Dental matrix protein-1 (DMP-1) present in eggshell, belongs to the family of the secretory calcium-binding phosphoproteins, some of which (like ovocleidin-116 and osteopontin) have been demonstrated to regulate eggshell calcification [9, 41]. Although DMP-1 has been extensively studied in association with tissue mineralization [41, 42], its effects on mineralization are still not characterized. Our previous data showed that the elevation of mammillary-knob density was associated with the increased expression of proteoglycan and glycoprotein genes in uterus, which contributed to the improvement of eggshell ultrastructure and mechanical properties in the late laying period [43]. However, we did not observe any modifications in mammillary-knob density and the concentration-dependent effects of glycoproteins on ultrastructure attributes need further investigation. Besides, protein modifications were reported to affect the interactions between calcium and matrix proteins, and affect the occlusions of proteins or organics inside the crystals [44]. The enriched term for protein modification process in the current study indicated that age-related co-translational or post-translational modifications of proteins (e.g., glycosylation and phosphorylation) might cause dysfunction of matrix proteins in shell formation. In summary, a disturbance of the expression of matrix protein could inevitably obstruct eggshell calcification, presumably contributing to the impaired shell ultrastructure and quality in aged hen group.

A decline of immune function is a common feature in aged animals. Immune hypofunction was also reported in reproductive tract of aged hens, evidenced by less frequency of immunocompetent cells (Bu-1^+^, CD3^+^, CD4^+^ and CD8^+^ cells), which can respond to bacterial and viral stimuli by releasing classical immune mediators [45–47]. In this study, the expression of BF2, YF5, MHCIA5 and MHCIA6, together with the GO terms in association with antigen processing and presentation, were all downregulated in aged hen group. MHC class I and II molecules present antigen peptides to CD8^+^ and CD4^+^ T cells, respectively, which can define the specificity of adaptive immune responses and determine the susceptibility to infectious pathogens [48]. BF2 is the single gene in chickens for classical peptide antigen presentation and MHC class I α-chain (YF) genes encoded molecules are capable of presenting antigen in a non-classical manner but similar to the classical class I molecules [49]. MHCIA5 and MHCIA6 were reported to be involved in the presentation of peptides derived from the endoplasmic reticulum lumen [50]. Therefore, the decreased expression of BF2, YF5, MHCIA5 and MHCIA6 with relevant downregulations of GO clusters of antigen processing and presentation might suggest a hypofunction of antigen processing and presentation, resulting in a compromised immune function and an elevated susceptibility to viral and bacterial infection in uterus of aged hen group. Analogously, age-related disorder in human immune system was associated with the dysfunction in antigen processing and presentation [51, 52]. These findings may provide clues for understanding age-related immune hypofunction of the reproductive tract in other poultry species and in mammals.

The compromised ability to mediate effective immune response against pathogens would elevate the risk of infection and trigger inflammation [53]. Higher contents of IL-1β in this study and the emigration of leukocytes previously reported [54] indicated an inflammation state in uterine mucosa of aged hens. Age-related inflammation characterized by high levels of proinflammatory cytokines has also been reported in bovine endometrial cells [55] and mammal intestinal mucosa [56]. Infection or inflammation could further stimulate the upregulations of proteins possessing antimicrobial defense [57]. The current data revealed that proteins in relation to immune defense, such as LOC107056420 (intestinal mucin-like protein) and ABCA13, were upregulated in aged hens as compared to young hens. Similarly, it was reported that aging induced increased expression of tight junction proteins (occludin) and antibacterial proteins (Avian β-defensins-1, 2, 10 and 11) in the uterine mucosa of laying hens [58]. In support of this view, higher concentrations of immune-defense proteins ovotransferrin and ovocleidin-17 were observed in eggshell from aged hens [21]. However, proteomic study on eggshell and uterine fluid suggested a negative relationship between eggshell quality and proteins involving in defense against bacterial [59]. The upregulation of these proteins in this study may have an adverse impact on eggshell quality. Furthermore, IL-1β has been demonstrated to disrupt the expression of eggshell formation-related proteins such as calcium-binding protein D28K (CaBP-D28K), presumably due to the roles of IL-1β in promoting protein degradation [60]. However, no difference was observed in the expression of calcium transporter and the calcium contents of eggshell between groups. This may be because aging is characterized by a state of chronic, low-grade, systemic inflammation with a moderate increase in IL-1β, which was not enough to cause perturbations of Ca^2+^ transport. In contrast, an intense increase in IL-1β induced by virus infection [61, 62] or uterus culture with 100 ng/mL recombinant chicken IL-1β [60] resulted in a disturbance of CaBP-D28K expression. Recent findings have indicated the important roles of proteoglycans in both immune response and eggshell formation [38, 63]. Age-related alterations in immune functions inevitably disturb the expression of proteoglycans in uterus and consequently disrupt eggshell formation. Therefore, age-related immune hypofunction might disrupt the expression of matrix proteins, probably contributing to the impaired eggshell quality in aged hen group.

Age-related increases in uterus lipid accumulation were regarded as an obstacle to uterine functions, since removal of lipid accumulation by induced molting could result in oviductal regression [64], presumptively favoring the improvement of ultrastructure and eggshell quality. In this study, DEGs including SCD, ACSBG1, APOC3 and FABP5, were mapped to PPAR signaling pathway, which has been considered as important sites for lipid metabolism [65]. It may suggest a disordered regulation of lipid metabolism in the uterus of aged hens, but the effects of these changes on uterine lipid deposition or eggshell formation need further investigation.

## Conclusions

This study suggested that decreased incidence of early fusion and increased thickness of mammillary layer are the crucial variations leading to the compromised mechanical properties of eggshell in the late laying period. A disturbed regulation of matrix protein expression and immune hypofunction in uterus of aged laying hens could contribute to age-induced ultrastructural deterioration of eggshell. These findings provide insights into the mechanism underlying age-related deterioration in eggshell ultrastructure, which may contribute to future studies on improving eggshell quality in the late laying period.

## Methods

### Animals and housing

The animal protocol for our study was approved by the Animal Care and Use Committee of the Feed Research Institute of the Chinese Academy of Agricultural Sciences. A total of 96 healthy young (42 wk. of age) and 96 healthy aged (72 wk. of age) Hy-Line Brown laying hens were individually divided into 8 replicates of 12 birds each in a randomized block design, and 3 birds were placed in one cage. The layer chicks were commercially obtained from Xiaoming Agriculture and Animal Husbandry Co. Ltd. (Ningxia, China) and were separately reared in two houses with similar configurations. Before this experiment, all birds used in this study were raised in one hen house to acclimate the environment for 4 wks. All the laying hens were fed with the same corn-soybean meal basal diet (Additional file 4) and provided with feed and water ad libitum with exposure to 16 h of light/d and the control temperature. All hens remained in good health during the feeding period. The egg production in young and aged hen groups were 95.05 ± 1.90% and 86.94 ± 0.49%, respectively. There were no culled birds, and medical intervention was not applied to any bird.

### Sample collection

A total of 24 egg samples (8 eggs/replicate/d × 3 d) from each replicate were collected on three successive days and weighed. Another 12 egg samples (4 eggs/replicate/d × 3 d) from each replicate were collected only for the measurement of stiffness, elastic modules and fracture toughness. An automatic-monitoring control system (FRI, CAAS, Beijing, China) was used to record the daily oviposition time and the total time an egg spent in the oviduct could be calculated. A total of 16 birds (8 replicates with 2 birds each) from each group were sacrificed at 8.5 h PO corresponding to the initiation stage of eggshell calcification. The rest of the birds were still raised until elimination. One sample from the aged hen group was rejected for further analysis, because no egg with incomplete shell was present in hen oviduct. The mucosa of uterine tissues surrounding the eggs were collected and snapped frozen immediately in liquid nitrogen, then stored at − 80 °C until further analysis.

### Eggshell physical and mechanical properties

Eggshell thickness was measured by the Egg Shell Gauge and breaking strength was determined by Egg Force Reader (Israel Orka Food Technology Ltd., Ramat Hashron, Israel). Shell stiffness was measured and the elastic modules and fracture toughness of each egg were calculated according to the formulas as previously described [66]. After removing egg contents, eggshell was washed, air-dried at room temperature and weighed. The shell ratio was calculated as shell weight/egg weight × 100.

### Eggshell ultrastructure

Three pieces of shell sample (~ 0.5 cm^2^) from the equatorial section of each egg were assessed for the ultrastructure of the external shell surface and the cross section by scanning electronic microscopy (SEM, FEI Quanta 600, Thermo Fisher Scientific Ltd., Portland, OR, USA). Shell specimens for the ultrastructure of mammillary layer were prepared according to previous methods [67, 68]. Mammillary thickness, the effective thickness (total thickness of palisade, vertical crystal layer and cuticle) and the width of the mammillary knobs were determined and calculated with SEM ruler. Mammillary knob density was counted and expressed as the number of knobs per unit. Each ultrastructural variant in the mammillary layer was assigned a score depending on its incidence in the eggshell. The total score was calculated by the sum of all variant scores [67, 68].

### Calcium and phosphorus contents in diets and eggshell

Diet and eggshell samples were dissolved in a solution containing HNO_3_ and H_2_O_2_ (v/v = 1:1) and then digested by the microwave dissolution instrument (MDS-10, Shanghai Xinyi Instrument Technology co., Ltd., Shanghai, China). Calcium and phosphorus contents were measured by flame atomic absorption spectrophotometry (Zeenit700P, Analytik Jena, Germany) and a spectrophotometer (UV-2700, Shimadzu, Japan).

### Extraction and determination of matrix protein in eggshell

Organic matrix proteins in eggshell were extracted and determined as previously described [69]. Eggshell powder (500 mg, pooled from 24 eggs from each replicate) was weighed and demineralized with 20% acetic acid. These samples were mixed with distilled water (v/v = 1:1) and freeze-dried. Then, they were dissolved in an extraction milieu (4 °C; about 12 h) and these extracts were dialyzed (cutoff 3500 Da). Each sample was centrifugated (20 min; 2500 g), and the supernatant was used to determine the protein content (PC0020; Beijing Solarbio Science & Technology Co., Ltd., Beijing, China).

### Proinflammatory cytokine contents in uterus tissue

The contents of IL-1β, IL-6 and IFN-α in uterus tissues were measured with ELISA kits (Shanghai Enzyme-linked Biotechnology Co., Ltd., Shanghai, China) as recommended by the manufacturer’s instructions. The results were normalized against total protein content in each sample for comparison.

### RNA extraction, library preparation and sequencing

Total RNA in the uterus tissues was extracted using TRIzol reagent according to the manufacturers’ instructions (Tiangen Biotech Co. Ltd., Beijing, China). Before library construction, the RNA integrity was assessed using the Agilent Bioanalyzer 2100 system (Agilent Technologies, CA, USA) with the RNA Nano 6000 Assay Kit. RNA samples (1 μg) were used for RNA library construction with NEBNext® Ultra™ RNA Library Prep Kit for Illumina® (NEB Inc., Ipswich, MA, USA). The construction involved mRNA purification, fragmentation under elevated temperature, and the synthesis of first and second strand cDNA. Exonuclease/polymerase were used for the conversion of the remainder overhangs into the blunt ends. The cDNA library construction included the adenylation of 3′ ends of DNA fragments, NEBNext Adaptor ligation and PCR. The purification of PCR products was conducted on AMPure XP system (Beckman Coulter, Beverly, USA) and the assessment of library quality was conducted on Agilent Bioanalyzer 2100 system (Agilent Technologies, CA, USA). After clustering, the library sequencing was carried out on an Illumina platform. The sequencing data have been deposited at Sequence Read Archive of National Center for Biotechnology Information (https://www.ncbi.nlm.nih.gov/bioproject; accession number: PRJNA627801).

### Sequence quality control and functional annotation of DEGs

Clean reads were obtained by removing raw reads of low quality, or with adapter and ploy-N. Q20, Q30, GC-content and sequence duplication level of the clean data were assessed. These clean reads were aligned to the reference genome (*Gallus gallus* 5.0; http://asia.ensembl.org/index.html) using Hisat2 tools soft. Function annotation was performed on the basis of the databases below: Nt (NCBI non-redundant nucleotide sequences; https://www.ncbi.nlm.nih.gov/), COG (Clusters of Orthologous Groups; http://www.ncbi.nlm.nih.gov/COG) and GO (Gene Ontology; http://geneontology.org). Fragments per kilobase of transcript per million fragments mapped (FPKM) were used to estimate the levels of gene expression. Differentially expression was analyzed with the DESeq2 and the resulting *P* values were adjusted using the Benjamini and Hochberg’s approach for controlling the false discovery rate (FDR). GO analysis of DEGs (FC > 1.5, FDR < 0.05) was conducted using the GOseq R packages based Wallenius non-central hyper-geometric distribution [70] and the KEGG pathway (http://www.genome.jp/kegg/) enrichment analysis of DEGs was performed using KOBAS software [71].

### qRT-PCR validation of RNA sequencing results

Eight genes were selected for qRT-PCR validation. The RNA samples were reverse transcribed with the FastQuant RT kit (KR106, Tiangen Biotech Co. Ltd., Beijing, China) to prepare cDNA. The mRNA expression of target genes was examined by qRT-PCR using CFX96 touch RT-PCR detection system (Bio-rad laboratories. Inc., CA, USA) with a 20 μL PCR reaction mixture (primer concentration: 0.3 μM) according to instructions of the SuperReal PreMix Plus kit (SYBR Green, FP205, Tiangen Co., Beijing, China). Primers used in this study are shown in Additional file 5. The reaction conditions were as follows: 95 °C for 15 min; 40 cycles of 95 °C for 10 s, 60 °C for 30 s. Each sample was measured in duplicate. The size of all amplified products was confirmed by electrophoresis on a 1.5% (w/v) agarose gel with gelred (SolarGelRed Nucleic Acid Gel Stain, Beijing Solarbio Science & Technology Co., Ltd., Beijing, China) and visualized in Gel Doc XR+ System (Bio-rad laboratories. Inc., CA, USA). The relative mRNA expression levels were normalized to avian β-actin by the 2^-ΔΔCt^ method [72].

### Statistical analysis

Unpaired t-tests (two tailed) were used to analyze the significant differences between groups using SPSS (version 23.0 for Windows; SPSS Inc., Chicago, IL, USA). Data were presented as mean with standard deviation (SD) and statistical significance was defined as a *P* value < 0.05.

## Supplementary Information

**Additional file 1.** Sequence quality and alignment information of uterus in young (42 wk. of age; T1–16) and aged hen groups (72 wk. of age; T17–31).

**Additional file 2.** The information of differentially expressed genes (DEGs) in uterus between young (42 wk. of age) and aged hen groups (72 wk. of age).

**Additional file 3.** Cluster of Orthologous Genes (COG) classification of differentially expressed genes of uterus in aged hen group (72 wk. of age) relative to young hen group (42 wk. of age).

**Additional file 4.** Ingredient and nutrient levels of the experimental diets (air-dried basis).

**Additional file 5.** Sequences for real-time PCR primers.

## Data Availability

The RNA-Seq datasets are available in the Sequence Read Archive of National Center for Biotechnology Information (https://www.ncbi.nlm.nih.gov/bioproject; accession number: PRJNA627801).

## Abbreviations

ACSBG1: Acyl-CoA synthetase bubblegum family member 1
APOC3: Apolipoprotein C3
BF2: MHC class I antigen BF2
CaBP-D28K: Calcium-binding protein D28K
CACNA1C: Calcium voltage-gated channel subunit alpha1 C
CACNA1D: Calcium voltage-gated channel subunit alpha1 D
CDH6: Cadherin-6
COG: Clusters of Orthologous Groups
DEGs: Differentially expressed genes
DMP1: Dental matrix protein-1
FABP5: Fatty acid binding protein 5
FC: Fold change
FDR: False discovery rate
FPKM: Fragments per kilobase of transcript per million fragments mapped
GO: Gene Ontology
GPC: Glypicans
KEGG: Kyoto Encyclopedia of Genes and Genomes
MHC: Major histocompatibility complex
MHCIA: MHC class I A molecules
Nt: NCBI non-redundant nucleotide sequences
PLCL1: Phospholipase C like 1
PO: Post-oviposition
PPAR: Peroxisome proliferator-activated receptor
RF: Rich factor
SCD: Stearoyl-CoA desaturase
SEM: Scanning electronic microscopy
VCAN: Versican
YF5: MHC class I α-chain
