# Supplementary material for: Uterine transcriptome analysis reveals mRNA expression changes associated with the ultrastructure differences of eggshell in young and aged laying hens
Source: BMC Genomics. 2020 Nov 9;21:770. doi: 10.1186/s12864-020-07177-7 (PMC7654033; doi:10.1186/s12864-020-07177-7)
Supplement: Supplementary file 1 — Additional file 1. Sequence quality and alignment information of uterus in young (42 wk. of age; T1–16) and aged hen groups (72 wk. of age; T17–31). [file 12864_2020_7177_MOESM1_ESM.docx]

**Additional file 1** Sequence quality and alignment information of uterus in young (42 wk of age; T1-16) and aged hen groups (72 wk of age; T17-31)

| Young hen group | | | | |  | Aged hen group | | | | |
| --- | --- | --- | --- | --- | --- | --- | --- | --- | --- | --- |
| Sample | Total reads | Mapped reads | GC content | Q30 |  | Sample | Total reads | Mapped reads | GC content | Q30 |
| T1 | 44,568,358 | 91.29% | 49.48% | 94.00% |  | T17 | 50,019,126 | 88.07% | 51.15% | 91.49% |
| T2 | 46,891,254 | 88.92% | 50.08% | 93.93% |  | T18 | 53,010,462 | 91.93% | 48.84% | 92.77% |
| T3 | 41,774,872 | 91.96% | 49.94% | 94.17% |  | T19 | 44,374,262 | 90.95% | 48.85% | 91.86% |
| T4 | 43,228,118 | 91.34% | 49.52% | 94.53% |  | T20 | 51,597,734 | 91.23% | 48.92% | 91.54% |
| T5 | 47,515,012 | 91.21% | 49.44% | 94.44% |  | T21 | 43,935,046 | 91.63% | 48.54% | 91.41% |
| T6 | 42,193,544 | 90.51% | 49.93% | 94.36% |  | T22 | 57,442,454 | 92.10% | 48.33% | 92.37% |
| T7 | 52,639,854 | 89.56% | 50.29% | 94.25% |  | T23 | 42,750,070 | 91.91% | 48.22% | 92.16% |
| T8 | 41,240,688 | 92.42% | 49.59% | 94.50% |  | T24 | 50,258,696 | 92.32% | 48.49% | 92.55% |
| T9 | 40,251,790 | 92.03% | 48.86% | 94.46% |  | T25 | 42,157,358 | 91.19% | 48.32% | 92.09% |
| T10 | 42,317,800 | 89.26% | 50.04% | 94.47% |  | T26 | 53,622,768 | 91.75% | 48.12% | 92.13% |
| T11 | 41,148,202 | 90.98% | 49.74% | 94.41% |  | T27 | 53,009,618 | 91.37% | 48.34% | 91.70% |
| T12 | 50,601,234 | 90.53% | 50.00% | 94.18% |  | T28 | 52,288,196 | 91.48% | 48.08% | 92.07% |
| T13 | 47,366,278 | 90.72% | 48.82% | 93.86% |  | T29 | 57,179,608 | 91.24% | 48.43% | 91.83% |
| T14 | 43,120,858 | 91.26% | 49.26% | 93.96% |  | T30 | 50,202,174 | 92.20% | 47.95% | 91.71% |
| T15 | 56,877,858 | 88.49% | 49.73% | 94.03% |  | T31 | 42.739.612 | 91.15% | 48.01% | 92.26% |
| T16 | 53,558,308 | 90.62% | 50.08% | 94.21% |  |  |  |  |  |  |

GC, guanine-cytosine; Q30, the proportion of bases with a Phred quality score greater than 30.

Data are from 8 replicates with 2 birds each (one sample from the aged hen group was rejected due to no egg with incomplete shell present in hen oviduct).
