# Supplementary material for: Uterine transcriptome analysis reveals mRNA expression changes associated with the ultrastructure differences of eggshell in young and aged laying hens
Source: BMC Genomics. 2020 Nov 9;21:770. doi: 10.1186/s12864-020-07177-7 (PMC7654033; doi:10.1186/s12864-020-07177-7)
Supplement: Supplementary file 3 — Additional file 3. Cluster of Orthologous Genes (COG) classification of differentially expressed genes of uterus in aged hen group (72 wk. of age) relative to young hen group (42 wk. of age). [file 12864_2020_7177_MOESM3_ESM.docx]

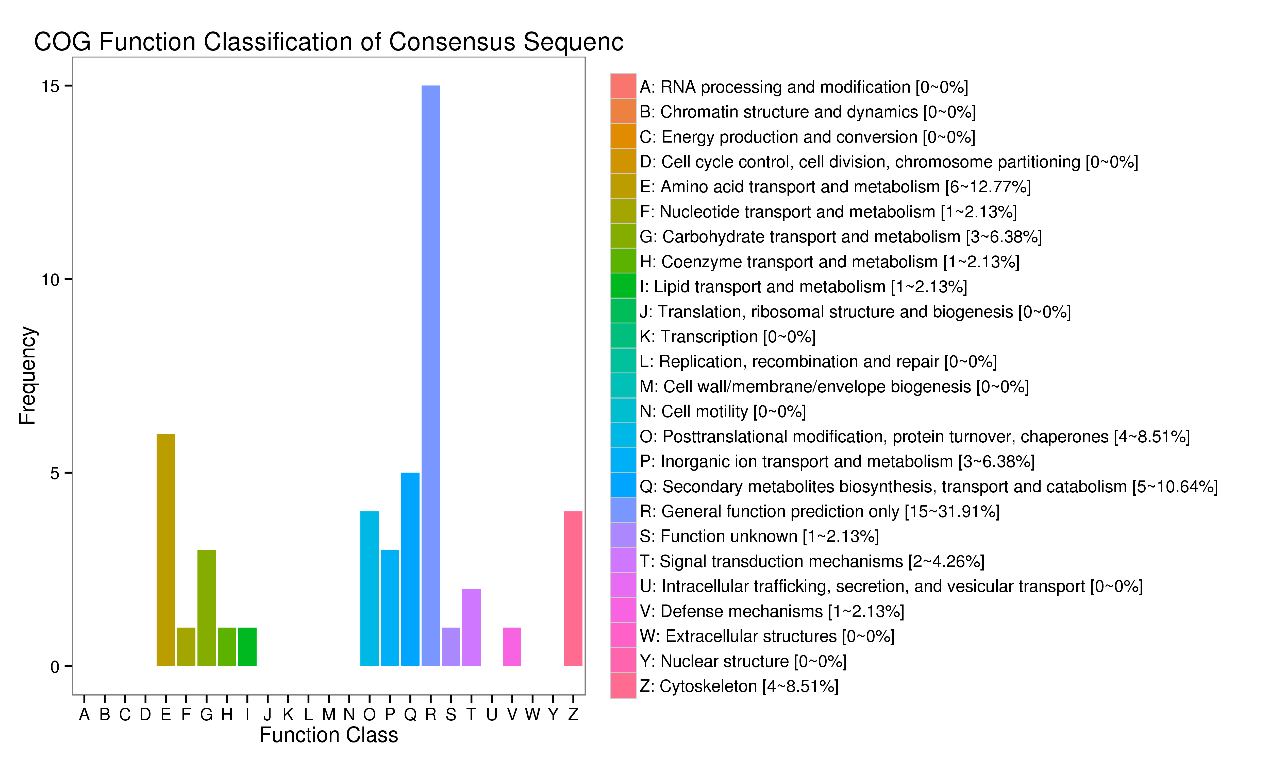


**Additional file 3** Cluster of Orthologous Genes (COG) classification of differentially expressed genes of uterus in aged hen group (72 wk of age) relative to young hen group (42 wk of age).
