# Supplementary material for: Uterine transcriptome analysis reveals mRNA expression changes associated with the ultrastructure differences of eggshell in young and aged laying hens
Source: BMC Genomics. 2020 Nov 9;21:770. doi: 10.1186/s12864-020-07177-7 (PMC7654033; doi:10.1186/s12864-020-07177-7)
Supplement: Supplementary file 4 — Additional file 4. Ingredient and nutrient levels of the experimental diets (air-dried basis). [file 12864_2020_7177_MOESM4_ESM.docx]

**Additional file 4** Ingredient and nutrient levels of the experimental diets (air-dried basis)

| Ingredient (%) |  | Nutrient levels |  |
| --- | --- | --- | --- |
| Corn | 65.20 | AME (MJ/kg)^1^ | 11.20 |
| Soybean meal | 23.80 | Crude protein (%) | 16.50 |
| Limestone | 8.60 | Methionine (%) | 0.35 |
| Salt | 0.30 | Lysine (%) | 0.78 |
| D,L-Methionine | 0.10 | Available phosphorous (%) | 0.39 |
| Dicalcium phosphate | 1.78 | Methionine + cysteine (%) | 0.60 |
| Choline chloride | 0.10 | Calcium (%)^3^ | 3.39 |
| Premix^2^ | 0.12 | Phosphorous (%)^3^ | 0.45 |
| Total | 100.00 |  |  |

^1^AME, apparent metabolism energy.

^2^Premix provided the following per kg of the diet: vitamin A, 12,500 IU; vitamin D_3,_ 4,125 IU; vitamin E, 15 IU; vitamin K, 2 mg; thiamine, 1 mg; riboflavin, 8.5 mg; calcium pantothenate, 11 mg; niacin, 32.5 mg; pyridoxine, 8 mg; biotin, 0.5 mg; folic acid, 1.25 mg; vitamin B_12,_ 0.02 mg; Mn, 65 mg; I, 1 mg; Fe, 60 mg; Cu, 8 mg; Zn, 66 mg; choline, 1,000 mg; phytase, 300 mg; montmorillonite, 1,000 mg; yeast culture, 10 g.

^3^Measured values.
