# Supplementary material for: Uterine transcriptome analysis reveals mRNA expression changes associated with the ultrastructure differences of eggshell in young and aged laying hens
Source: BMC Genomics. 2020 Nov 9;21:770. doi: 10.1186/s12864-020-07177-7 (PMC7654033; doi:10.1186/s12864-020-07177-7)
Supplement: Supplementary file 5 — Additional file 5. Sequences for real-time PCR primers. [file 12864_2020_7177_MOESM5_ESM.docx]

**Additional file 5** Sequences for real-time PCR primers

| Name | Forward sequence (5’-3’) | Reverse sequence (5’-3’) | Accession no. |
| --- | --- | --- | --- |
| BF2 | AGAGGCAGTTCCCACCAAGAGG | CAGCCACTCCACGCAGGTTTC | NM_001031338.1 |
| SLC9A9 | TCGCACTGGCTATTCAAGACACAG | GCCTCCACCAAATACCCAGACTG | NM_001031305.2 |
| MHCIA6 | TGGAAGAGCAAGTCAGGGTAGGAG | TGAAGCAGCACAGCACTCAGATG | XM_025155901.1 |
| YF5 | CGTTGGATTGGTGGTGTGGAAGAG | CAGCACAGCACTCAGATGGAAGG | NM_001030675.2 |
| VCAN | AGGCATTGTATCGCTGGCTGTTG | TTCAGTTGCTCTGGGCTTGCTATG | NM_204787.1 |
| CDH6 | CAGATGGGCGGATTATCAGGAACC | TGGTGAGTCAGGTGGCGTAGAC | NM_001001758.2 |
| OVAL | TTCCTGGGTAGAAAGTCAGACAAAT | GACAATGGCATTAACCAGAACCA | NM_205152.1 |
| PLCL1 | CGGCAGACATCACTCACGACAAG | GCACTGTGATATTCCACGCAAAGC | XM_421916.6 |

BF2, MHC class I antigen BF2; SLC9A9, solute carrier family 9 member A9; MHCIA6, major histocompatibility complex, class I, A6; YF5, MHC class I α-chain; VCAN, versican; CDH6, cadherin-6; OVAL, ovalbumin; PLCL1, phospholipase C like 1.
